# Use of the internet related to infertility by infertile women and men in Turkey

**DOI:** 10.12669/pjms.332.12620

**Published:** 2017

**Authors:** Duygu Gulec Satir, Oya Kavlak

**Affiliations:** 1 Dr. Duygu Gulec Satir, Research Assistant, Obstetrics and Gynecology Nursing Department, Ege University Faculty of Nursing, Izmir, Turkey; 2 Prof. Oya Kavlak, Obstetrics and Gynecology Nursing Department, Ege University Faculty of Nursing, Izmir, Turkey

**Keywords:** Internet, Infertility, Information needs, Health, Patients

## Abstract

**Objective:**

To determine differences in use of the Internet related to infertility between infertile women and men, whether they benefit or are negatively affected from information on the Internet, and share this information with health professional.

**Methods:**

This cross-sectional study was carried out with 285 infertile women and 158 men between December 2015 and February 2016. Data were collected by a survey Form which included questions related to sociodemographic characteristics, related to infertility (duration of treatment, type of treatment) and questions about use of the Internet. Chi-square analysis was used to evaluate the differences in Internet use and independent variables for patients.

**Results:**

Seventy-four percent of women and 68.4% of men used the Internet related to infertility. Women and men most often looked for information related to assisted reproductive technology and the causes of infertility. Men searched for information related to fertility drugs used in treatment significantly less than women. They often visited the websites of fertility centers and doctors. A high percentage of them have benefited from information on the Internet. Almost half of the women and men shared the information obtained from the Internet with health professional.

**Conclusion:**

Most frequently, infertile patients use the Internet to obtain information related to infertility and they benefited from information on the Internet. For health professional it is important to direct Internet users to safe and true information resources.

## INTRODUCTION

Infertility is an important health problem that has physical and emotional effects that concern 8–10% of women and men of reproductive age.^1^,^2^ In Turkish society, childless individuals have lesser status.^3^ They also experience many psychological problems such as loneliness, stigmatization, depression, social isolation, and infertility treatment process adversely affects their quality of life.^4^-^8^

In our society, they tend to hide the problem of infertility from social environment in order not to get negative reactions from the individuals around them or to be exposed to their intense questions and repressions.^9^-^11^ Therefore they need information throughout the complex infertility treatment.^1^ Need for information related to infertility causes infertility patients to use the Internet.^12^-^14^

The rate of Internet use related to infertility in studies conducted in other countries ranges from 55.8% to 81%.^14^-^16^ In Turkey, no other study has examined use of the Internet by infertile women and men to research health information relating to infertility. In this context, this study was performed to determine differences between infertile women and men in Turkey about rate and reasons for use of the Internet related to infertility, whether they benefit or are negatively affected from information on the Internet, and shared this information with health professional. It is thought that findings to be obtained as a result of this study will be instructive for health care providers during the counseling and training provided to infertility patients.

## METHODS

This cross-sectional study was carried out with women and men receiving infertility treatment at the Infertility and In-Vitro Fertilization Center of Tepecik Training and Research Hospital located in Izmir, the third-largest city in Turkey. The Tepecik Training and Research Hospital is the only in vitro fertilization center in the Aegean region affiliated with the Ministry of Health. Approximately 100 infertile couples are receiving assisted reproductive technology (ART) monthly.

A total 520 patients who received infertility treatment between December 2015 and February 2016 (3 months) were included in this study. Ultimately, research was carried out with 443 individuals because 24 were illiterate, 18 had Syrian origin and could not speak Turkish, and 35 refused to participate in the research.

### Questionnaire

Research data were collected in the waiting room before treatment through a survey form, which was developed from the relevant literature^15^-^17^ questions related to sociodemographic characteristics and questions related to infertility (duration of treatment, type of treatment); and questions about use of the Internet were asked.

The validity of the questionnaire was obtained by asking expert opinion of four university lecturers in gynecology and obstetrics nursing departments. The experts evaluated the questions as appropriate. Then, the questionnaire was tested for ease of understanding by giving it to 10 infertile patients who were not included in the study, and a changes were made based on their recommendations. An extra choice was added about ‘complementary and alternative medicine (CAM)’ for question related to the Internet use purpose. It was then used for data collection with patients.

### Ethic

Participation in the survey was voluntary. For research, the institution’s permission was obtained; infertility patients willing to participate were adequately informed about the purpose of the study, and their informed consent was obtained. Prior to commencing the study, approval was obtained from the Ethics Committee of Ege University Nursing Faculty (Reference number: 2015-141).

### Statistical analysis

Data were analyzed using the Statistical Package for the Social Sciences for Windows (SPSS for Windows, client version 16.0). Discrete variables were expressed as percentages and presented as frequency tables. The chi-square test and the independent t-test were used to identify significant differences between the women and men. Chi-square analysis was used to evaluate the differences in Internet use and independent variables for women and men.

## RESULTS

Women had a mean ± SD age of 31.3±5.3 years and men with a mean ± SD age of 33.5±5.7 years. Infertile women more likely to be unemployed (p<0.001), longer marriage (p<0.05) than men. Infertile women were receiving more IVF-ET treatment (p<0.05) than men, otherwise both groups were comparable. The demographic characteristics of the women and men are shown in Table-1.

**Table-I:** Sosyodemografic characteristics of infertile women and men

*Demografic characteristics*	*Women n(%)*	*Men n(%)*	*p*
***Educational status***			
Literate but no formal education	9(3.2)	2(1.2)	
Primary school	82(28.8)	43(27.2)	
Moderate school	65(22.8)	35(22.2)	0.746
High school	85(29.8)	51 (32.3)	
University	44(15.4)	27 (17.1)	
***Employment status***			
Employed	93(32.6)	151 (95.6)	<0.001
Unemployed	192(67.4)	7 (4.4)	
Duration of marriage (years)	6.77 ±4.4	5.93 ±3.9	0.047
***Have children***			
Yes	17(6.0)	16 (10.4)	0.110
No	268(94.0)	142 (89.6)	
Duration of infertility (years)	5.7 ±4.3	5.0 ±3.8	0.093
***Type of Treatment***			
Insemination(IUI)	76(26.6)	56 (35.5)	
IVF-ET	200(70.2)	92 (58.2)	0.027
Intracytoplasmic sperm injection(ICSI)	9(3.2)	10 (6.3)	

Of the total 285 infertile women, 74.0 % used the Internet for infertility. This rate was 68% for men (p>0.05). Infertile women and men used the Internet for infertility-related purposes once or twice a month (51.7% and 65.7%; *p<*0.05), mostly searched to ART and the causes of infertility, and visited the websites of IVF-ET centers and doctors. Infertile men searched for information about only fertility drugs significantly less than women(*p<*0.05) and men visited the academic institution websites significantly more than women *(p<*0.01*)* (Table-II).

**Table-II:** Distribution of infertile women and men according to the Internet use.

	*Women n(%)*	*Men n(%)*	*P*
***Infertility-related the Internet use***		
Yes	211(74.0)	108(68.4)	0.202
No	74(26.0)	50(31.6)
***Frequency of the Internet use***		
Every day	22(10.4)	5(4.7)	0.034
1–2 times a week	80(37.9)	32(29.6)
1–2 times a month	109(51.7)	71(65.7)
Topics that they searched on the Internet*			
Causes of infertility	117(55.5)	50(46.3)	0.121
Infertility tests and treatments	96(45.5)	46(42.6)	0.621
Fertility drugs	69(32.7)	20(18.5)	0.008
ART	126(59.7)	62(57.4)	0.692
Communicate with other infertile people	36(17.1)	15(13.9)	0.464
CAM	25(16.8)	14(14.6)	0.647
			
Websites*			
Personal webpages of doctors	102(48.3)	45(41.7)	0.258
Academic institution websites	20(9.5)	21(19.4)	0.012
Infertility center websites	111(52.6)	46(42.6)	0.09
Online-forum websites	47(22.3)	32(29.6)	0.150

* More than one item could be indicated.

When the relationship between Internet use and sociodemographic characteristics were examined, women and men that had a higher education level, employed and without children used the Internet more often. In addition, women who received IVF-ET treatment used the Internet more often *(p<*0.05*)*. (Table-III).

**Table-III:** The relationship between the Internet use and other variables in infertile women and men.

*Variables*	*Women*		*Men*	

	*Who use n (%)*	*Who do not use n (%)*	*Who use n (%)*	*Who do not use n (%)*
***Educational status***				
Moderate school and less	96(61.5)	60(38.5)	42(52.5)	38(47.5)
High school and above	115(89.1)	14(10.9)	66(84.6)	12(15.4)
	p<0.001		p<0.001	
***Employment status***				
Employed	81 (87.1)	12(12.9)	107(70.9)	44(29.1)
Unemployed	130(67.7)	62(32.3)	1(14.3)	6(85.7)
	p<0.001		p<0.01	
***Have children***				
Yes	9(52.9)	8(47.1)	7(43.7)	9(56.3)
No	202(75.4)	66(24.6)	101(71.1)	41(28.9)
	p<0.05		p<0.05	
***Type of Treatment***				
Insemination	47(61.8)	29(38.2)	36 (64.3)	20 (35.7)
IVF-ET	157(78.5)	43(21.5)	66 (71.7)	26 (28.3)
Intracytoplasmic sperm injection	7(77.8)	2(22.2)	6 (60.0)	4 (40.0)
	p<0.05		p:0.538	

The majority of women (83.9%) and men (78.7%) stated that they had benefited from information they found on the Internet. A minority of women (14.2%) and men (10.3%) were affected negatively. Women said it caused unhappiness (63.3%) (Table-IV).

**Table-IV:** Status of benefit and negative effect from information on the Internet.

	*Womenn (%)*	*Menn (%)*	*p*
***I benefited from information on the Internet***			
Yes	177(83.9)	85 (78.7)	0.253
No	34(16.1)	23 (21.3)	
What kind of benefit?*			
To obtain information	161(90.9)	74 (87.1)	Not compared
Seeking treatment	30(16.9)	23 (27.1)	
Choice of clinic	23(13.0)	9 (10.6)	
Compliance with their treatment process	31(17.5)	12 (14.1)	
Drug use	16(9.0)	4 (4.7)	
Social or emotional support	22(12.4)	9 (10.6)	
***I was affected negatively from information on the Internet***				
Yes	30(14.2)	7 (10.3)	0.102
No	181(85.8)	101 (89.7)	
What kind of negative effect?*			
Hopelessness	19(63.3)	2 (28.6)	Not compared
Confusing	3(10.0)	2 (28.6)	
Panic	2(6.6)	1(14.3)	
Misdirection	5(16.5)	1 (14.3)	
Despondence	1(3.4)	1(14.2)	

* More than one item could be indicated.

About half of women and men (52.6% and 56.5%) shared the information obtained with health professional; 28.4 % of women and 30.6% of men had searched the information after checkups.

## DISCUSSION

The present study has established that about three quarters of infertile women used the Internet for this research and men used less than women. This rate in studies conducted in other countries was 55.8% in Canada, 54% in Holland, and 54% in the UK.^14^-^16^ Compared to our research, these reduced rates are thought to be related to the date research was conducted. Studies were carried out between 1997 and 2002. Considering the rapidly increasing Internet use over time, the rate of Internet use in our study is expected to be higher than other study findings.

About half of the women and two-thirds of men had used the Internet just once or twice a month. In a similar study, most of the couples (67%) used it once a month or more often; in another study, 53.1% used it once a month or less.^14^-^15^ Research findings show similarity. Also in the present study, as a different finding, men used the Internet significantly less frequently than women.

Infertile women and men have most often searched for information on topics such as ART, causes of infertility, and infertility-related tests and treatment. Rawal and Haddad (2005) reported that the Internet was most frequently used for general information related to infertility problems and treatment.^14^ Weissman et al., (2000) stated that 80% of infertility patients searched for information about diagnosis and treatment of infertility on the Internet; 51% have searched for clinics.^16^ According to Haagen et al. (2003), the leading topics were IVF-ET, infertility, endometriosis, pregnancy, ICSI, and fertility.^15^ Infertile individuals search similar topics on the Internet. However in the present study, as a different finding, men searched for information about fertility drugs significantly less than women. It is thought that reason of this circumstance is men used fertility drugs in treatment less than women.

Infertile women and men have most often visited websites of fertility clinics and doctors, and forum sites. Similarly, in other studies, infertile individuals have most often visited health-related websites, academic institutions’ and fertility clinics’ websites.^15^-^17^ In addition to these sites, Alghamdi and Moussa (2012) reported that 51.6% of patients sought information on doctors’ sites.^18^ The reason infertility patients visit these sites is thought to be because they trusted clinics and doctors. In the present study, as a different finding, men used academic institution websites significantly more than women.

Infertile women and men who have attained higher education levels, employed and without children used the Internet more often. In similar studies also, those who have higher education levels used the Internet to search for information related to health.^15^,^17^,^18^ Infertile individuals who have children may use the Internet related infertility more than others; because they demonstrated fertility. Also in the present study women who are receiving IVF-ET treatment used the Internet more often. It is thought that if the process of treatment getting more complex, women use the Internet increasingly.

In the present study, a majority of women and men reported that they benefited from the information obtained from the Internet, and they said that the Internet helped them obtain information, seek treatment, and adjust to the treatment process. Similarly, in Haagen et al.’s study (2003), infertile individuals reported that Internet usage has developed the information related to infertility and facilitated the decision-making process about therapy.^15^ Weissman et al., (2000) suggested that 30% of patients took advantage of the Internet during the decision-making process.^16^ It can be said that infertility patients usually benefit from Internet use. In the present study a small percentage of women and men said that the information obtained from the Internet had negatively affected them. The unsuccessful experiences shared especially in the forum sites can be said to influence infertility patients adversely.

About half of women and men shared the information with health professional; however, nearly one third of women and men stated that they later searched on the Internet for information they had talked about in their infertility clinics. In a similar study, 17% of infertile couples shared the information they found on the Internet with health professional, and 36% said they were encouraged by health professional to use the Internet.^15^ AlGhamdi and Moussa (2012) reported that 72.5% of patients shared their information with doctors.^18^ Infertility requires a complex treatment process that affects couples’ lives. In this process, individuals use the Internet as a secondary source besides health professionals.

## CONCLUSION

It was established at the end of our research that women used the Internet for infertility- related issues more than men. Similarly, women and men have used the Internet to obtain information related to infertility such as the causes of infertility, ART. They generally have benefited from the data on the Internet. Infertility is a period that causes stress for couples and when they need information. It is recommended that health professionals who provide care to infertile individuals should provide conducive environment for discussion to correct wrong information.

### Authors’ Contribution

**DGS & OK:** Conceived, designed, did data collection, statistical analysis and manuscript writing. The authors alone are responsible for the content and writing of this article.
